# Melatonin, a potent agent in antioxidative defense: Actions as a natural food constituent, gastrointestinal factor, drug and prodrug

**DOI:** 10.1186/1743-7075-2-22

**Published:** 2005-09-10

**Authors:** Rüdiger Hardeland, SR Pandi-Perumal

**Affiliations:** 1 Institute of Zoology and Anthropology, University of Göttingen, Berliner Str. 28, D-37073 Göttingen, Germany; 2 Comprehensive Center for Sleep Medicine, Department of Pulmonary, Critical Care and Sleep Medicine, Mount Sinai School of Medicine, 1176 - 5^th ^Avenue, New York, NY 10029, USA

## Abstract

Melatonin, originally discovered as a hormone of the pineal gland, is also produced in other organs and represents, additionally, a normal food constituent found in yeast and plant material, which can influence the level in the circulation. Compared to the pineal, the gastrointestinal tract contains several hundred times more melatonin, which can be released into the blood in response to food intake and stimuli by nutrients, especially tryptophan. Apart from its use as a commercial food additive, supraphysiological doses have been applied in medical trials and pure preparations are well tolerated by patients. Owing to its amphiphilicity, melatonin can enter any body fluid, cell or cell compartment. Its properties as an antioxidant agent are based on several, highly diverse effects. Apart from direct radical scavenging, it plays a role in upregulation of antioxidant and downregulation of prooxidant enzymes, and damage by free radicals can be reduced by its antiexcitatory actions, and presumably by contributions to appropriate internal circadian phasing, and by its improvement of mitochondrial metabolism, in terms of avoiding electron leakage and enhancing complex I and complex IV activities. Melatonin was shown to potentiate effects of other antioxidants, such as ascorbate and Trolox. Under physiological conditions, direct radical scavenging may only contribute to a minor extent to overall radical detoxification, although melatonin can eliminate several of them in scavenger cascades and potentiates the efficacy of antioxidant vitamins. Melatonin oxidation seems rather important for the production of other biologically active metabolites such as *N*^1^-acetyl-*N*^2^-formyl-5-methoxykynuramine (AFMK) and *N*^1^-acetyl-5-methoxykynuramine (AMK), which have been shown to also dispose of protective properties. Thus, melatonin may be regarded as a prodrug, too. AMK interacts with reactive oxygen and nitrogen species, conveys protection to mitochondria, inhibits and downregulates cyclooxygenase 2.

## Introduction

In several countries, melatonin is sold over the counter; in others its free sale is prohibited. The usefulness of melatonin as a food additive continues to be a matter of debate. Meanwhile, countless people have used melatonin for mitigating the symptoms of jet lag, an application which has been tested and is recommended [1-4]; any person we have spoken to has reported positive experiences. Melatonin has been and is being used in several clinical trials with different therapeutic approaches. In some of these studies, in addition to improvements of sleep, the repeatedly demonstrated antioxidant properties [5-10] were the main reason for testing the pineal hormone. This holds especially for the treatment of neurodegenerative disorders, such as Alzheimer's disease [11-13] and amyotrophic lateral sclerosis [14].

In terms of application it seems necessary to thoroughly analyze the mechanisms of antioxidant actions of melatonin and to distinguish between effects observed at pharmacological or physiological concentrations. These considerations must not be restricted to the melatonin released from the pineal gland into the circulation and to the classic hepatic degradation route of 6-hydroxylation followed by conjugation. On the contrary, we would like to lay emphasis on the significance of tissue melatonin and the alternate oxidative pathways of catabolism leading to different, biologically active products. The relationship between melatonin and nutrition will be discussed, with regard to the presence of the compound as a natural food constituent sometimes affecting circulating levels, to the post-prandial release of melatonin from the gastrointestinal tract, and to interactions with other antioxidants present in food. Finally, a model of mitochondrial protection is reviewed.

## Melatonin in food and in the gastrointestinal tract

Melatonin is a natural compound of almost ubiquitous occurrence [15-17]. Its presence was demonstrated in all major taxa of organisms, as far as tested, including bacteria, unicellular eukaryotes, macroalgae, plants, fungi and invertebrate animals. Several studies dealt with melatonin in edible plants [8,18-25]. One can conclude that relevant quantities of melatonin are present in most vegetables, fruit, nuts and cereals. However, the precise melatonin contents are sometimes affected by some uncertainties which result from particular methodological problems arising in material from photoautotrophic organisms. First, melatonin can be easily destroyed by oxidants during extraction [26], and, second, false positive and false negative data are easily obtained due to the presence of secondary plant metabolites, either mimicking melatonin or interfering with it in the assays [16,17,21,22]. It is a strict requirement to apply preservative conditions of extraction, to control the yield by determinations of recovery, and to obtain data by two methodologically different procedures. Although this has not been done in any plant tested, the widespread occurrence of melatonin in plants is beyond doubt. To date, the presence of melatonin was demonstrated in more than 20 dicot and monocot families. Usually, the amounts of melatonin reported varied considerably between species and between plant tissues, from the detection threshold to several hundred pg/g fresh weight. One should, however, be aware that these concentrations frequently greatly exceed avian and mammalian blood levels, which rarely attain more than 200 pg/mL during the nocturnal maximum, and can remain below 10 pg/mL during the day. Intestinal resorption of dietary melatonin should not be a particular problem because the amphiphilic molecule can easily cross any membrane. Therefore, an efficient uptake of the indoleamine from food should be expected to influence the blood plasma concentration (see below). Melatonin was observed to be elevated in alpine and mediterranean plants exposed to strong UV irradiation [25], a finding which may be seen in relation to melatonin's antioxidant properties antagonizing damage by light-induced oxidants. It is particularly worth mentioning the very high levels reported for several seeds and medicinal plants [8,15,24,27,28] (Table 1). The high amounts frequently found in seeds may be interpreted in terms of antioxidative protection within a dormant and more or less dry system, in which enzymes are poorly effective and cannot be upregulated, so that low molecular weight antioxidants such as melatonin are of advantage [20]. Moreover, melatonin's amphiphilicity may favor its accumulation especially in oily seeds.

**Table 1:** Particularly high melatonin levels reported for several edible and medicinal plants (selected examples).

**Species**	**Tissue**	**Melatonin [ng/g]**	**References**
***(A) Edible plants***			
*Lycopersicon esculentum *(tomato)	fruit	0.5	[18]
*Raphanus sativus *(red radish)	root tuber	0.6	[19]
*Brassica campestris *(Japanese radish)	stem, leaves	0.6	[19]
*Brassica nigra *(black mustard)	seed	129	[24,28]
*Brassica hirta *(white mustard)	seed	189	[24,28]
*Prunus cerasus *(tart cherry, Montmorency)	fruit	15–18	[23,24]
*Prunus amygdalus *(almond)	seed	39	[28]
*Pimpinella anisum *(anise)	seed	7	[24,28]
*Foeniculum vulgare *(fennel)	seed	28	[24,28]
*Helianthus annuus *(sunflower)	seed	29	[24,28]
*Oryza sativa *(rice)	seed	1	[19]
*Zea mays *(Indian corn)	seed	1.3	[19]
*Avena sativa *(oat)	seed	1.8	[19]
*Festuca arundinacea *(tall fescue)	seed	5	[19]
*Elettaria cardamomum *(green cardamom)	seed	15	[24,28]
*Zingiber officinale *(ginger)	tuber	0.5	[19]
*Musa paradisiaca *(banana)	fruit	0.5	[18]
			
***(B) Officinal plants***			
*Melissa officinalis *(balm mint)	young plant	16	[25]
*Scutellaria baicalensis *(huang-qin)	plant	> 2,000 – > 7,000	[24,25,27]
*Pimpinella peregrina *(-)	dried root	38	[25]
*Hypericum perforatum *(St. Johns wort)	leaf	1,750	[27]
*Hypericum perforatum *(St. Johns wort)	flower	> 2,400 – > 4,000	[25,27]
*Lippia citriodora *(lemon verbena)	young plant	22	[25]
*Tanacetum parthenium *(feverfew)	leaf (fresh/dried)	> 1,300/> 7,000	[24,25,27]

In some of the medicinal plants, interactions or synergisms of melatonin with secondary metabolites may be of importance. In *Scutellaria baicalensis*, e.g., melatonin is accompanied by acteoside, baicalein, baicalin, wogonin and ganhuangenin, substances with antioxidant, antiinflammatory, sedating and immunomodulatory properties, interfering also with NO synthases and P_450 _monooxygenases, i.e., functions within the action spectrum of melatonin or affecting melatonin metabolism [17]. Melatonin is also present in fungi and, with regard to nutrition, this may be relevant especially for yeast. In cultures freshly prepared from commercially available cubes of baker's yeast, μmolar concentrations of melatonin were measured, sometimes exceeding 40 μM [29,30].

It is not yet known whether food is the only external source of melatonin in mammals. The presence of melatonin in bacteria including *Escherichia coli *[31] may suggest a contribution by intestinal bacteria to the high amounts of the indoleamine found in the gut [cf. discussion in ref. [17]]. However, strictly anaerobic bacteria, which predominate in the colon, have not yet been investigated.

The gastrointestinal tract deserves particular attention, not only with regard to melatonin uptake, but, even more, as an extrapineal site of melatonin biosynthesis, where this molecule is present in amounts exceeding those found in the pineal gland by several-hundred-fold, and from where it can be released into the circulation in a post-prandial response, especially under the influence of high tryptophan levels [32-36]. Gastrointestinal melatonin is also released to the lumen and participates in enterohepatic cycling [37-39]. Therefore, nutrition is not only linked to melatonin by uptake, but also by the influences of other food constituents and digestive physiology on melatonin release.

With regard to nutrition, a decisive question is whether the amounts of melatonin present in the food can suffice for changing its level in the blood plasma. This was first indicated by findings of Hattori *et al*. [19], who observed rises in plasma melatonin after feeding plant material rich in this compound. However, this result allowed a different interpretation, because other substances including its precursor tryptophan might have elicited a post-prandial release of gastrointestinal melatonin. This argument was recently refuted, at least in chicken, because the removal of melatonin from feed caused decreases in plasma levels [40]. The result gave rise to a statement that melatonin may not only be regarded as a hormone and a tissue factor, but also, in a sense, as an antioxidant vitamin.

The redox properties of melatonin may be unfavorable for its preservation in the food. Being an easily oxidizable compound capable of directly detoxifying several free radicals and other oxidants, leads, in turn, to the consequence of non-enzymatic destruction. The experience with melatonin extraction from plant material lets us assume that only a certain fraction of the compound present in food will arrive in the gut and even less in the circulation. Nevertheless, it may be possible that melatonin metabolites, especially substituted kynuramines formed by oxidative pyrrole-ring cleavage, which also possess protective properties and sufficient amphiphilicity [41-43], and/or their derivatives are taken up from the food and will turn out to be beneficial.

## Reactions of melatonin with oxidants

With regard to the presence of melatonin in food, in medicinal plants and to the use as a food additive, its antioxidant and other protective properties deserve attention. Since the discovery of melatonin oxidation by photocatalytic mechanisms involving free radicals [15,44,45], scavenging by this indoleamine has become a matter of particular interest. Melatonin was also shown to be oxidized by free radicals formed in the absence of light [46], and its capability of scavenging hydroxyl radicals at high rates [47-51] initiated numerous investigations on radical detoxification and antioxidative protection. Melatonin turned out to be considerably more efficient than the majority of its naturally occurring structural analogs [47,50-52], indicating that the substituents of the indole moiety strongly influenced reactivity and selectivity. Rate constants determined for the reaction with hydroxyl radicals were in the range between 1.2 × 10^10 ^and 7.5 × 10^10 ^M^-1 ^s^-1^, depending on the methods applied [53-57]. Regardless of differences in the precision of determination, melatonin has been shown, independently by different groups, to be a remarkably good scavenger of this radical species. This property can be crucial for antagonizing oxidative damage under pharmacological and other *in vitro *conditions. To what extent this may contribute to physiological protection remains, however, a matter of debate.

Meanwhile, melatonin has been shown to react with many other oxidants, such as carbonate radicals [58-60], singlet oxygen [15,34,61-65], ozone [15,34], and several biologically occurring aromatic radicals, such as protoporphyrinyl and substituted anthranilyl radicals [15,59,61,62,66,67]. Reactions with other non-biological radicals were also described [15,34], among which the ABTS cation radical [ABTS = 2, 2'-azino-*bis*-(3-ethylbenzthiazoline-6-sulfonic acid)] merits special attention because of its analytical value. This extremely long-lived radical which is stable for many days provides a good example for single-electron donation by melatonin [52,68]. This conclusion was unambiguously confirmed by cyclic voltammetry [69]. Single-electron donation is important for several reasons. Free radicals can react with scavengers in different ways, either by abstraction of an electron, or a hydrogen atom, or by addition. In the case of melatonin, radical addition has been observed or predicted theoretically only for interactions with hydroxyl radicals [69-72] and nitric oxide [69,73-75]. Electron/hydrogen abstraction, however, is a common key step for interactions of melatonin with oxidizing free radicals of both high and low reactivity and, therefore, reflects melatonin's property as a broad spectrum antioxidant. Electron abstraction was also concluded to be a primary step of melatonin oxidation in a pseudoenzymatic reaction catalyzed by oxoferrylhemoglobin [76]. Single-electron transfer reactions are also believed to play a role in detoxification of resonance-stabilized free radicals, such as carbonate and aryl radicals, which are frequently underrated in their destructive potential because of their lower reactivity, compared to the hydroxyl radical. However, due to their longer life-time they can reach more distant sites than the extremely short-lived hydroxyl radical, which exists only for nanoseconds. The capability of melatonin of scavenging carbonate and certain aryl radicals may be of much higher significance and protective value than previously thought. Finally, according to a recently proposed model, single-electron exchange is thought to be the basis for interactions of melatonin with the mitochondrial respiratory chain [77,78] which is assumed to require only very small, quasi-catalytic amounts of melatonin and which would convey antioxidative cell protection by radical avoidance rather than detoxification of radicals already formed (see below).

Reactive nitrogen species represent another category of potentially destructive substances, which react with melatonin. Scavenging of nitric oxide by melatonin in a nitrosation reaction is well documented [9,79-81]. Whether this can be regarded as a detoxification reaction keeping NO from forming the more dangerous peroxynitrite is uncertain because nitrosomelatonin easily decomposes, thereby releasing NO [82], an experience also made with other NO adducts from respective scavengers including NO spin traps [83]. Scavenging of peroxynitrite has also been described [9,80,81,84], although it is sometimes difficult to distinguish betweeen direct reactions with peroxynitrite and with hydroxyl radicals formed by decomposition of peroxynitrous acid. What seems more important than direct scavenging of peroxynitrite is the interaction with products from the peroxynitrite-CO_2 _adduct (ONOOCO_2_^-^), namely, carbonate radicals (CO_3_•^-^) and •NO_2 _[79,85]. In the presence of bicarbonate/CO_2_, this pathway is favored and the primary interaction of melatonin is that with CO_3_•^- ^[85], a conclusion in agreement with results from other studies on CO_3_•^- ^scavenging [58-60]. The mixture of CO_3_•^- ^and •NO_2 _represents the physiologically most efficient nitration mixture, because of the high availability of CO_2 _in biological material. It is worth noting that melatonin can, in fact, decrease 3-nitrotyrosine levels, as shown in guinea pig kidney [86].

Another highly interesting aspect of melatonin's antioxidant actions, which may be particularly important from the nutritional aspect, is its interactions with classic antioxidants. In both chemical and cell-free systems, melatonin was repeatedly shown to potentiate the effects of ascorbate, Trolox (a tocopherol analog), reduced glutathione, or NADH [50,68,69,87]. These findings, which can be clearly distinguished from additive effects, surprisingly indicate multiple interactions via redox-based regeneration of antioxidants transiently consumed. This may, in fact, be of practical importance, since melatonin was also shown to prevent decreases in hepatic ascorbate and α-tocopherol levels *in vivo*, under conditions of long-lasting experimental oxidative stress induced by a high cholesterol diet [88].

## Metabolites of melatonin, a scavenger cascade, and melatonin as a prodrug

**Figure 1:**
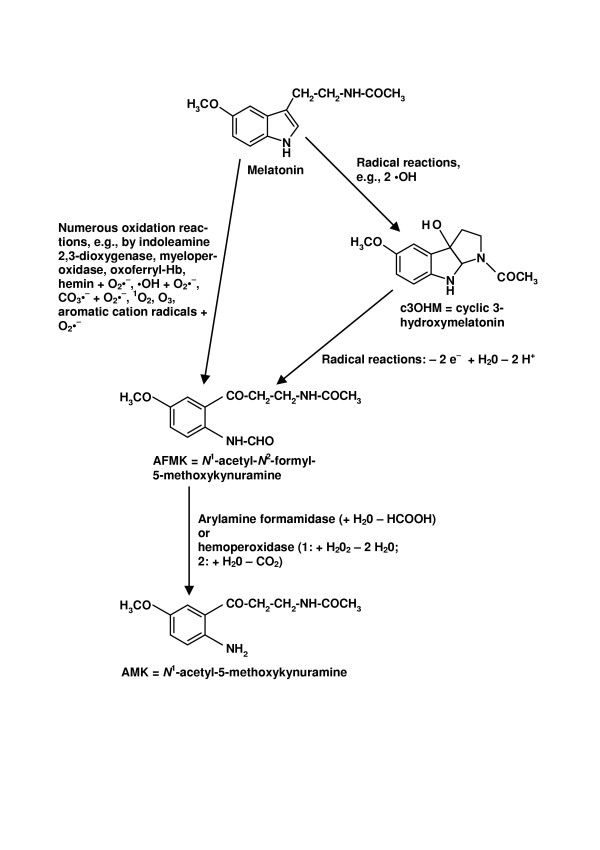
The kynuric pathway of melatonin metabolism.

Reactions of melatonin with free radicals and other oxidants are not only a matter of the toxic reactants eliminated, but also of the products formed. It is highly important to distinguish between metabolites formed under physiological or near-physiological conditions from those produced in chemical systems designed for studying reactions with a single radical species in preparations as pure as possible. Disregard of this point has led to several misinterpretations in the past. We have repeatedly emphasized that studies using reaction systems which preferentially generate hydroxyl radicals mainly lead to hydroxylated adducts or their derivatives such as substituted indolinones, whereas biological material usually contains orders of magnitude more superoxide anions than hydroxyl radicals. Therefore, an entirely different product spectrum is obtained as soon as hydroxyl radicals, or other electron-abstracting radicals, act in the presence of an excess of superoxide anions [60,89]. Radicals derived from melatonin by interaction with a first, reaction-initiating radical likely combine with superoxide anions so that the radical reaction chain is readily terminated [15,49]. The product formed by oxidative pyrrole-ring cleavage is a substituted kynuramine, *N*^1^-acetyl-*N*^2^-formyl-5-methoxykynuramine (AFMK; Fig. 1). We have investigated numerous reaction systems and in all those containing sufficient quantities of superoxide anions, AFMK was by far the most abundant product [44,46,58-60,66,89]. Interestingly, a profound and sursprising difference exists between melatonin and other structurally related indoleamines. While substituted kynuramines represent only a limited or small fraction of oxidation products from other indolic compounds, AFMK usually greatly exceeds the total of other substances formed. This indicates a significant contribution not only of the 5-methoxy residue, but also of the *N*-acetylated side chain to the oxidation chemistry of melatonin, a conclusion corroborated by various scavenging assays and chemiluminescence associated with pyrrole-ring cleavage [52]. Moreover, AFMK was the only melatonin metabolite detected in culture media of various aquatic organisms, unicells and small metazoans, whereas several additional products were found in axenic media incubated for extended periods of time [90]. AFMK formation seems to be a favored pathway of melatonin degradation in these species.

These findings do not represent a peculiarity of non-vertebrates, but rather seem to reflect the non-hepatic melatonin catabolism in vertebrates. Contrary to statements in the earlier literature claiming that almost all melatonin is metabolized in the liver to 6-hydroxymelatonin followed by conjugation and excretion, recent estimations attribute about 30 percent of overall melatonin degradation to pyrrole-ring cleavage [91]. The rate of AFMK formation may be considerably higher in certain tissues, since extrahepatic P_450 _monooxygenase activities are frequently too low for a high turnover via 6-hydroxylation. The high amounts of gastrointestinal melatonin (see above), as far as they are not released unmetabolized, have to enter a pathway different from monooxygenation. AFMK formation is highly likely.

The significance of pyrrole-ring cleavage in oxidative metabolism of tissue melatonin is particularly illustrated in the central nervous system, where a secondary product, *N*^1^-acetyl-5-methoxykynuramine (AMK) derived from AFMK by deformylation, was identified as a main metabolite [92]. When melatonin was injected into the *cisterna magna*, about 35 percent was recovered as AMK. Under the conditions used, AFMK and AMK were the only products formed from melatonin in the brain and no 6-hydroxymelatonin was detected. In this case, the high turnover in the kynuric pathway of melatonin catabolism is the more remarkable as it cannot be explained on the basis of the enzymes capable of catalyzing the formation of AFMK: (i) indoleamine 2, 3-dioxygenase which uses tryptophan as the main substrate, exhibits sufficiently high activities only after inflammatory stimulation of the microglia [93-95]; (ii) myeloperoxidase, which can also catalyze pyrrole-ring cleavage of melatonin [91,96,97], is again associated with activated phagocytes. To assume free radical reactions as the main cause of kynuric melatonin degradation in the brain is, therefore, highly suggestive. Non-enzymatic AFMK formation in other tissues will be a matter for future research.

It is a remarkable fact that AFMK is formed by many different mechanisms [summarized in refs. [15,41,59,66,89]]. Apart from the enzymes mentioned, pseudoenzymatic catalysis by oxyferrylhemoglobin or by hemin, interactions with free radicals, e.g., combinations of •OH and O_2_•^-^, or CO_3_•^- ^and O_2_•^-^, or organic cation radicals and O_2_•^-^, oxidation by singlet oxygen, by ozone, or by O_2 _under photoexcitation of melatonin all lead to AFMK. Even another product formed from melatonin by interactions with free radicals, cyclic 3-hydroxymelatonin [70], can be further metabolized by free radicals to AFMK [68]. All these findings indicate that AFMK is a central metabolite of melatonin oxidation especially in non-hepatic tissues.

As already mentioned, AFMK is easily deformylated to AMK. To date two enzymes capable of catalyzing this reaction have been identified, arylamine formamidase and hemoperoxidase [49,89,98]. The two methoxylated kynuramines, AFMK and AMK, are of particular interest because of their own radical-scavenging and protective properties. In any case, kynuramines, a separate class of biogenic amines, exhibit various biological activities [99], which are, however, rarely investigated. With regard to antioxidative protection, AFMK was shown to reduce 8-hydroxy-2-deoxyguanosine formation [42] and lipid peroxidation, and to rescue hippocampal neurons from oxidotoxic cell death [41]. Although AFMK interacts, not surprisingly, with the highly reactive hydroxyl radicals, it is otherwise relatively inert towards radicals of lower or intermediate reactivity [43,89]. This is convincingly explained by its preference for two-electron transfer reactions as demonstrated by cyclic voltammetry [41].

The deformylated product AMK, easily formed from AFMK [92], appears to be a highly interesting substance, for several reasons: first, it is a radical scavenger of considerably higher reactivity than AFMK because it easily undergoes single-electron transfer reactions [43,89,100] and, second, it acts as a cyclooxygenase (COX) inhibitor that is much more potent than acetylsalicylic acid [101] and has relative specificity for COX-2 (B Poeggeler, pers. commun.). Moreover, AMK was recently shown to downregulate COX-2 expression in macrophages [102]. AMK might, therefore, contribute to the attenuation of oxidative stress both directly and indirectly by interference with inflammatory responses. A third, mitochondrial effect will be discussed below. Unfortunately, the precise tissue levels of AMK are still unknown, partially because of a lack of specific assays, partially due to its high reactivity which readily leads to other products. Since AMK can be recovered from the urine after a melatonin load [92], sufficient amounts may be present in the tissues, at least after administration of pharmacological doses. Therefore, melatonin seems to act not only directly, but, additionally, as a prodrug of AMK.

It is a remarkable fact that the kynuric pathway of melatonin metabolism includes a series of radical scavengers, which may be regarded as a scavenger cascade [68], with a possible sequence of melatonin → cyclic 3-hydroxymelatonin → AFMK → AMK, where melatonin can be alternately converted to AFMK directly. From melatonin to AFMK, up to 4 free radicals can be consumed [68]; recent determinations [Rosen J, Hardeland R, unpubl. data] have shown that even higher numbers of free radicals can be eliminated, and other, previously unknown products are being characterized. The potent scavenger AMK consumes further radicals in primary and secondary reactions. Interestingly, AMK not only interacts with reactive oxygen but also with reactive nitrogen species and several products have been structurally characterized in Göttingen [[103]; manuscript in preparation]. Neither the end of the kynuric pathway of melatonin nor that of the scavenger cascade is in sight.

## Multiple levels of antioxidative protection by melatonin

**Figure 2:**
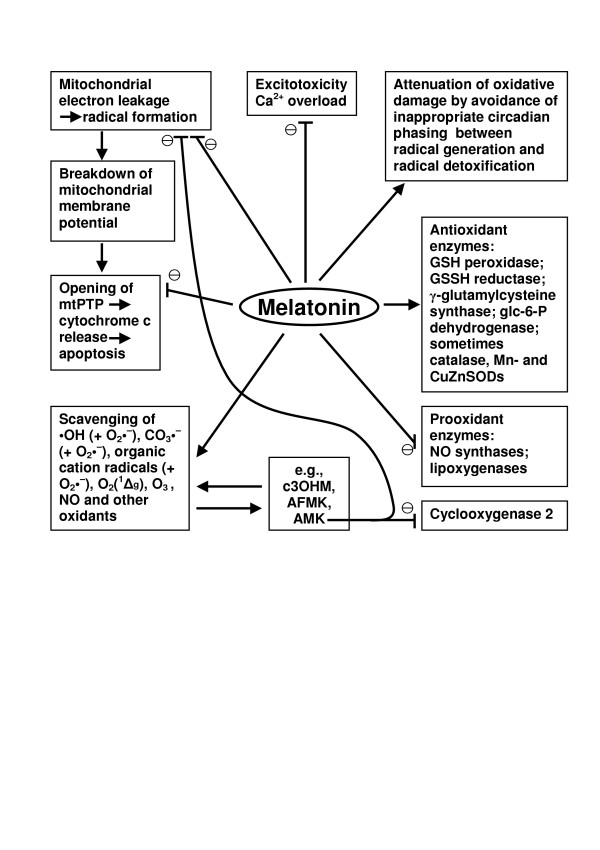
Overview of the pleiotropic actions of melatonin and some of its metabolites in antioxidative protection.

Antioxidative protection by melatonin is not just a matter of direct radical scavenging (Fig. 2), as becomes immediately evident from stoichiometry. Although tissue levels of melatonin can be considerably higher than those in the circulation, the quantities of free radicals generated in its metabolism would still be too high for the available amounts of the indoleamine. Our understanding is that direct scavenging by physiological concentrations of melatonin by a non-enzymatic contribution to the kynuric pathway and the subsequent actions of the metabolites formed becomes important. Signaling effects of melatonin, however, are always possible at physiological levels.

Melatonin upregulates several antioxidant enzymes. Most frequently, this has been demonstrated for glutathione peroxidase [7,77,88,104-118] and sometimes glutathione reductase [7,108,112,119], presumably indirectly via GSSG. In some tissues Cu, Zn- and/or Mn-superoxide dismutases [7,108-112,117-123] and, rarely, catalase [112,118,123,124] are upregulated. Stimulation of glutathione peroxidase seems to be widely distributed among tissues and is observed quite regularly in both mammalian and avian brain; upregulations in other organs were more variable. The action of melatonin on glutathione metabolism seems to exceed the effects mentioned. Stimulation of glucose-6-phosphate dehydrogenase [108] and γ-glutamylcysteine synthase [10,112] indirectly supports the action of glutathione peroxidase by providing reducing equivalents (NADPH) for the action of glutathione reductase and by increasing the rate of glutathione synthesis, respectively.

Contrary its effect on the enzymes of glutathione metabolism, the effect of melatonin on superoxide dismutase subforms and catalase strongly depends on organs and species. Stimulation was observed in some tissues, but not in others; in some cases, even decreases were reported. This may not only be a matter of differences in responsiveness of cell types. The complexity in the regulation of the respective enzymes has to be considered. Frequently, they exhibit compensatory rises in response to oxidative stress. When melatonin is counteracting experimentally induced stress, the result may be a normalization of enzyme activity, i.e., lower values, compared to animals treated with oxidotoxins, rather than inductions. Such normalizations were, in fact, described [114,125]. However, in cases of stronger oxidative stress, active centers of enzymes may be destroyed by the free radicals generated and normalization of enzyme activities by melatonin administration appears as an increase [110,124,125].

An additional aspect of melatonin's actions on antioxidant enzymes deserves future attention: In two neuronal cell lines, physiological concentrations of melatonin not only induced glutathione peroxidase and superoxide dismutases at the mRNA level, but concomitantly increased the life-time of these mRNAs [117].

Melatonin also contributes to the avoidance of radical formation in several independent ways. It downregulates prooxidant enzymes, in particular 5- and 12-lipoxygenases [112,126-128] and NO synthases [9,34,77,108,112,129-134]. The widely observed attenuation of NO formation is particularly important in terms of limiting rise in the strongly prooxidant metabolite peroxynitrite and of the free radicals derived from this compound, namely, •NO_2_, carbonate (CO_3_•^-^) and hydroxyl (•OH) radicals. Suppressions of both lipoxygenase and NO synthase may additionally set limits to inflammatory responses, although the immunomodulatory actions of melatonin are certainly more complex and may involve additional effects of melatonin and AMK, too.

Another widely unexplored but potentially important signaling effect of melatonin in antioxidative protection concerns quinone reductase 2 [77,135,136]. This enzyme, which is implicated in the detoxification of potentially prooxidant quinones, binds melatonin at upper physiological concentrations, so that it had originally been presumed to represent a melatonin receptor. Although its precise function under the influence of melatonin is not yet fully understood, the relationship to the indoleamine may become of future interest from the standpoint of nutrition, since quinones are taken up with food, especially, vegetables.

Although less relevant from a nutritional point of view, melatonin also contributes indirectly to radical avoidance, e.g., by its antiexcitatory effects in the central nervous system, and as an endogenous regulator molecule controlling rhythmic time structures. This last action may be particularly important for well-timed alimentary melatonin supplementation in the elderly, who exhibit a strongly reduced amplitude in the circadian melatonin rhythm. The significance of appropriate timing for maintaining low levels of oxidative damage has been overlooked for quite some time. However, temporal perturbations as occurring in short-period or arrhythmic circadian clock mutants lead to enhanced oxidative damage, effects observed in organisms as different as *Drosophila *and the Syrian Hamster [77,137,138].

In the last few years, mitochondrial effects of melatonin have been discovered which may turn out to be even more important than the protective actions described above. Mitochondria are the main source of free radicals in the majority of animal cells and are implicated in aging processes. The importance of mitochondrial diseases is increasingly perceived. Mitochondria play a key role in apoptosis. Notably, several of the mitochondrial effects of melatonin were obtained at low pharmacological doses in drinking water [116,139,140] or even at near-physiological concentrations down to 1 nM [141].

Several studies of mitochondrial effects revealed attenuation of mitochondrial lipid peroxidation, prevention of oxidative protein and DNA modifications, preservation of ultrastructure, resistance against toxins etc., findings which were widely in line with previous concepts of protection [10,113,142-146]. Moreover, melatonin was shown to affect redox-active compounds in mitochondria, in particular, to decrease NO [143,147] and to restore normal levels of reduced glutathione [113,144] and coenzyme Q_10 _[148].

More importantly, beyond these rather conventional findings, with few exceptions, melatonin was found to increase mitochondrial respiration and ATP synthesis, in conjunction with rises in complex I and IV activities [112,141-143,146,147,149,150]. Complex I and IV activities were also found to be increased by melatonin in hepatic mitochondria of senescence-accelerated mice [116,140,151]. Moreover, melatonin was found to enhance gene expression of complex IV components [147].

The improvements of ATP formation and O_2 _consumption are presumably not decisive for protection, but can serve as good indicators for the reduction of electron leakage from the respiratory chain. Electron transfer to molecular oxygen at the matrix side, largely at iron-sulfur cluster N2 of complex I [152], is a major source of free radicals. This process also diminishes electron flux rates and, therefore, the ATP-generating proton potential. Processes affecting the mitochondrial membrane potential such as calcium overload, either due to overexcitation, to protein misfolding or to damage by free radicals, are antagonized by melatonin. In cardiomyocytes, astrocytes and striatal neurons, melatonin prevented calcium overload [153,154], counteracted the collapse of the mitochondrial membrane potential induced by H_2_O_2 _[153], doxorubicin [155] or oxygen/glucose deprivation [154], and also inhibited the opening of the mitochondrial permeability transition pore (mtPTP), thereby rescuing cells from apoptosis. In addition to the antioxidant actions, melatonin directly diminished mtPTP currents, with an IC_50 _of 0.8 μM [154], a concentration which would require mitochondrial accumulation of melatonin, something which is possible again due to the amphiphilicity of melatonin.

**Figure 3:**
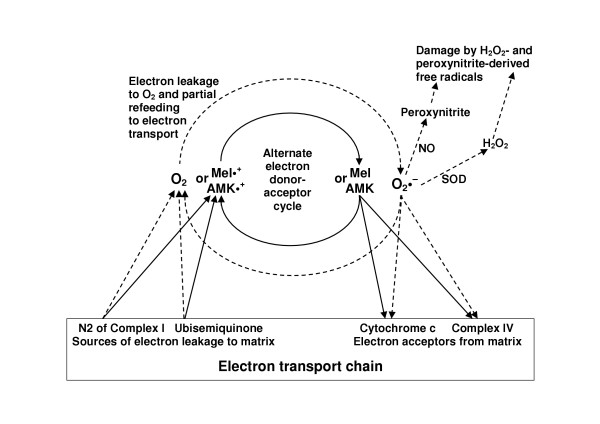
A model of mitochondrial radical avoidance and support of electron flux by melatonin and its metabolite AMK. The potent electron donors melatonin and AMK are thought to feed electrons into the respiratory chain, thereby forming resonance-stabilized cation radicals which may efficiently compete with molecular oxygen for electrons leaking from iron-sulfur cluster N2 or from ubisemiquinone. The competition reduces superoxide anion formation and, thereby, the generation of secondary radicals; at the same time, electrons re-fed to the electron transport chain contribute to the maintenance of the proton potential and, thus, to ATP synthesis. The model is partially hypothetical, but might explain observations of reductions in electron leakage and oxidant formation as well as an enhancement of ATP formation.

The effects of melatonin on the respiratory chain open new perspectives for diminishing radical formation, instead of seeking only antioxidant effects for the elimination of radicals already formed. We have proposed a model of radical avoidance (Fig. 3) in which electron leakage is reduced by single-electron exchange reactions betwen melatonin and components of the electron transport chain [77,78]. In fact, mitochondrial H_2_O_2 _formation was found to be reduced by melatonin [156]. The basic idea of the model is that of a cycle of electron donation to the respiratory chain, eventually to cytochrome c [78], followed by reduction of the formed melatonyl cation radical by electron transfer from N2 of complex I. The cation radical is assumed to act as alternate electron acceptor competing with molecular oxygen, thereby decreasing the rate of O_2_•^- ^formation. In addition to the electrons being largely recycled, most of the melatonin is also. Therefore, such a mechanism would only require very low, quasi-catalytic amounts of melatonin, in accordance with the effects demonstrated with nanomolar concentrations. Because the recycled electrons are not lost for the respiratory chain, this would also lead to improvements in complex IV activity, oxygen consumption and ATP production. Alternately, the melatonin metabolite AMK, which is also highly reactive and can undergo single-electron transfer reactions [43], may act in the same way. The prediction of our model of mitochondrial protection by AMK was confirmed by other investigators [147]: AMK was shown to exert effects on electron flux through the respiratory chain and ATP synthesis very similar to those observed with melatonin.

A highly attractive aspect of mitochondrial protection results from the small quantities required: experimentally induced mitochondrial damage in rat fetuses was even prevented by maternally administered melatonin [146]. The mechanism as outlined, requiring only low amounts of melatonin or its metabolite AMK, would make these compounds even more interesting from a nutritional point of view. The amounts present in selected food, such as some vegetables, but even more nuts and cereals, could suffice for maintaining tissue levels of the indoleamine capable of safeguarding mitochondrial function, particularly in elderly persons whose nocturnal melatonin maxima in pineal gland and circulation have substantially declined with age. Transient moderate rises in blood melatonin during the day resulting from direct uptake or postprandrial release from the gastrointestinal tract should not be regarded as a problem in terms of circadian timing. The circadian system responds to melatonin according to a phase response curve [157,158]: the so-called silent zone, during which no substantial phase shifts are induced, extends throughout the largest part of the day.

## Safety of melatonin

Can all these findings on antioxidant and radical-avoiding actions of melatonin justify its intake as a food additive or as a medication? The idea of substitution therapy may seem especially attractive for the elderly who have more or less lost the nocturnal peak of circulating melatonin. Nevertheless, the use as a food additive is still a matter of controversy. The argument for a naturally occurring compound, which is a normal food constituent, cannot suffice alone, since commerical preparations would always lead to at least transient pharmacological concentrations in the blood, and the immunomodulatory actions of melatonin may not be desired in every case. Therefore, experience will have to answer the question of its usefulness. Without any doubt, melatonin is remarkably well tolerated. Of course, one can find in any large statistical sample of melatonin users some individuals who complain about side effects, scientifically understandable or not. In a currently running study on ALS, patients receiving daily very high doses of melatonin (30 or even 60 mg per day), we did not see any harmful side effects [14] and have not to date. In patients with rheumatoid arthritis, some symptoms were suspected to be associated with immunomodulatory actions of melatonin [159], so that caution is due in this group of individuals. More research will be required on melatonin in different diseases and disorders, but there is no good reason to assume that melatonin, at moderate or even at high doses, is dangerous to a healthy person or to patients with types of oxidative stress phenomena not caused by (auto-)-immune responses. One might also suspect that melatonin could exert unfavorable effects by increasing the blood pressure, due to downregulation of NO synthase and NO scavenging by the indoleamine itself or by AMK. Melatonin was tested in clinical trials on hypertension and was reported to decrease blood pressure in one study [160], but to interfere with nifedipine [161], whereas a combination of lacidipine with melatonin was recommended in another investigation [162]. Therefore, interaction with other medication has to be considered.

Problems of dosage and side effects may also arise from impurities in the melatonin preparations sold over the counter. Contaminants have repeatedly been detected in such material, including our own experience of that kind. As long as the contaminant is only AFMK, this may be less serious, but one should be aware that the pharmacology of kynuramines is only partially known. Moreover, manufacturers must consider that an easily oxidizable compound like melatonin can undergo reactions under air exposure. On large surfaces, such as silica gels, we see this every day in the laboratory.

Another important aspect for the use of melatonin as a food additive is timing. As soon as the substance is given as a pill or as a preparation from a medicinal plant causing relatively high pharmacological blood levels, the situation is entirely different from the uptake with normal food or from the postprandial gastrointestinal release. Since circulating melatonin peaks at night, pharmaceutical preparations should be strictly given at the same time of day in the evening. The usual recommendation „at bed time" may be insufficient since this could mean in practice different hours of the day. Here, one has to consider the chronobiological functions of melatonin. When given during the day, a high dose of melatonin would cause mild narcotic effects, drowsiness etc. and the practice is not recommended for this reason. It would not shift the circadian oscillator much, because of the silent zone of the phase response curve for melatonin, in which phase shifts are negligibly small. This is the same reason that a postprandial release of gastrointestinal melatonin does not shift the circadian oscillator. Advance shifts of the endogenous clock by melatonin are much larger at late afternoon and early night [157,158]. Therefore, melatonin should be given relatively precisely at the same hour, to avoid phase shifts differing in extent and pushing of the circadian oscillator back and forth. As mentioned above, pertubations of the internal time structure can also cause oxidative stress [77].

## Conclusion

In terms of nutrition, melatonin is interesting both as a natural constituent of food, and as a food additive. Its use for the latter purpose can be recommended only with some caution, given the present state of our knowledge, although the risks by melatonin appear remarkably low, compared to other medications and food additives. Melatonin's antioxidant capacity is based not only on direct radical detoxification, but comprises manifold effects. Some of the most promising areas, modulation of mitochondrial metabolism by melatonin and actions of its kynuric metabolites, deserve particular attention in the future and may change our view of the value of these compounds profoundly.

## List of abbreviations

ABTS: 2, 2'-azino-*bis*-(3-ethylbenzthiazoline-6-sulfonic acid)

AFMK: *N*^1^-acetyl-*N*^2^-formyl-5-methoxykynuramine

ALS: amyotrophic lateral sclerosis

AMK: *N*^1^-acetyl-5-methoxykynuramine

c3OHM: cyclic 3-hydroxymelatonin

COX-2: cyclooxygenase 2

GSSG: oxidized glutathione

## Competing interests

Authors declare that they have no competing interests concerning the use of melatonin or melatonin-containing preparations as a food additive.

## Authors' contributions

This review was initiated by SRP-P; a first version by RH was jointly revised.
